# A study on genotype–environment interaction based on GGE biplot graphical method in sunflower genotypes (*Helianthus annuus* L.)

**DOI:** 10.1002/fsn3.1610

**Published:** 2020-05-06

**Authors:** Isa Ansarifard, Khodadad Mostafavi, Mahmood Khosroshahli, Mohammad Reza Bihamta, Hosein Ramshini

**Affiliations:** ^1^ Department of Agronomy and Plant Breeding Science and Research Branch Islamic Azad University Tehran Iran; ^2^ Department of Agronomy and Plant Breeding, Karaj Branch Islamic Azad University Karaj Iran; ^3^ College of Agriculture & Natural Resources (UCAN) University of Tehran Karaj Iran; ^4^ College of Agriculture & Natural Resources University of Tehran Pakdasht Iran

**Keywords:** combined analysis of variance, main components, mega‐environment, sunflower

## Abstract

GGE biplot technique is one of the appropriate methods for investigating the genotype–environment interaction. An experiment was conducted to examine and evaluate the stability and adaptability of grain yield of 12 sunflower genotypes using the randomized complete block design (RCBD) with three replications in five regions including Karaj, Birjand, Firooz‐Abad, Kashmar, and Arak within two agricultural years. Analysis of variance indicated that the effect of location, year, location × year, genotype, and genotype × location was significant at 1% probability level. Results of biplot analysis showed that the first and second principle components accounted 50.6% and 22.8%, respectively, and in total 73.4% of grain yield variance. In this study, genotype, location, year, year × location, genotype × location, genotype × year, and genotype × year × location explained 2.75%, 17.36%, 5.47%, 17%, 10.8%, 1.04%, and 7.48% of total variance, respectively. Investigating the polygon view led to the identification of three top genotypes and also three mega‐environment. The first mega‐environment included Karaj, Birjand, and Kashmar. The second was Arak, and the third was Firooz‐Abad. To study the kernel yield and stability of genotypes simultaneously, average coordinate view of environments was used and it was determined that genotype *Zaria* with the highest grain yield has high yield stability also. Ranking the cultivars based on the ideal genotype introduced the genotype *Zaria* as the best genotype. The highest grain yield belonged to *Zaria* cultivar at 3.34 t/ha followed by *Favorite* with 3.23 t/ha. Results obtained from ranking the environments based on the ideal environment introduced Kashmar and Birjand regions as the best environments. Examining the biplot figure for testing environments correlation confirms the positive correlation among Karaj, Birjand, and Kashmar. Correlation between Karaj with Arak, Karaj with Firooz‐Abad, and Arak with Firooz‐Abad was negative. Arak and Firooz‐Abad were highly discriminating and representative and would be used to identification of superior genotypes.

## Graphical abstract

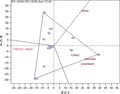

## 1 INTRODUCTION

Sunflower (*Helianthus annuus* L.) an annual plant, with cross‐pollination (Bhardwaj, 2009) from Asteraceae family, is a main source of edible oil production along with soybean, canola, cotton, and peanut around the world, and after the cotton and soybean, it is ranked as the third one in Iran (FAS, 2013;Saffari, 2006). Sunflower is widely consistent with different weather conditions and is slightly sensitive to the light period (Hu, Seiler, & Kole, 2010). The cultivated area of sunflower across the world is 24.6 million hectares, and its grain production is 36.4 million ton (FAS, 2013). The cultivated area of sunflower in Iran is 70,000 hectares, and grain production is 78,000 t (Anonymous, 2012;FAO, 2012). Depending on the varieties of sunflower, grain contains 26%–50% oil. Not only does not contain harmful fatty acids, but also contains beneficial fatty acids such as oleic acid, palmitic acid, and stearic acid (Seiler, 2007).

Genotype × environment interaction is very important for breeders, and it is considered one of the complicated issues of breeding program for preparing the high‐yielding and stable genotypes (Gauch, 2006;Yan, Kang, Ma, Woods, & Cornelius, 2007). The criterion for using various types of stability analysis techniques depends on the experimental design, plant, researcher's viewpoint, environment of experiment, and other conditions (Fattahi & Yossefi, 2006). A genotype's yield in the environment is affected with main effect of environment, main effect of genotype and genotype × environment interaction. Although the effect of the environment explains a large percentage of the total variation and the effects of genotype × environment is smaller, these two effects are involved in genotype evaluation and when selecting the top genotypes, the effect of the genotype and the genotype‐environment interaction should be considered together (Yan, 2002). Even so, there is a gap between the yields obtained by farmers and those obtained in experimental stations using the best management practices (Fischer & Edmeades, 2010). Characteristics such as grain yield and oil content in sunflower are complex and are determined by genetic, environmental, and genotype by environment interactions (Leon, Andrade, & Lee, 2003). Yan et al. (2007) believed that GGE biplot method is more successful than the AMMI technique in terms of data analysis of genotype–environment interaction. It is only the effect of genotype and genotype–environment interaction that are important in selecting the stable genotypes and the important point is that both effects of genotype and genotype–environment interaction should be examined simultaneously. GGE biplot technique makes possible to evaluate simultaneously and graphically these two effects (Yan & Kang, 2003). Existence of interaction effect of genotype and environment indicates that the best genotype in an environment is likely not the best genotype in other environments (Perkins & Jinks, 1971).

To estimate the genotype–environment interaction and determine the stable genotypes, numerous statistical techniques (univariate and multivariate) were used. Although the calculation and use of parametric and nonparametric univariate statistical techniques are easy, these methods cannot interpret properly the complicated and multidimensional nature of interaction. Hence, use of multivariate techniques is used to fix this problem (Moreno‐Gonzalez, Crossa, & Cornelius, 2004). GGE biplot method provides an excellent solution to combine the average of yield and stability and transform them into a criterion so that it can be evaluated graphically. GEI makes ambiguous the determination of accurate contribution of bred cultivars and the improved environment or technology in yield (Haruna et al., 2017;Muller et al., 2009;Roy, 2000).

In a study conducted to investigate the genotype and environment interaction on the sunflower cultivars in five locations, the Progress genotype was identified as the stable and high‐yielding genotype. Following the genotype Progress, genotypes Record and Gabur had the most stability and yield (Khomari, Mostafavi, & Mohammadi, 2017). To determine stability and reaction of sunflower genotypes to different environments, 10 sunflower cultivars were investigated in six regions of Iran. Using GGE biplot graphical method, the average of grain yield and genotypes’ stability were evaluated and genotypes Lakumka and Bulg3 had the most and the least yields, respectively. Based on the GGE biplot graphical method, the ideal genotype was diagnosed, that is, Lakumka. Two mega‐environments were identified in this experiment. Generally, using the GGE biplot graphical method in this research showed that utilizing this technique obtain new information which cannot be diagnosed by other methods and such technique is highly capable to investigate the stability of cultivars (Kalatejari, Mostafavi, & Nabipour, 2016). In particular, the grain‐filling phase has been reported to be highly variable between years, locations, and sowing dates due to the variations of temperature and solar radiation (Izquierdo et al., 2009). In an experiment conducted on ten canola cultivars in four locations, Mostafavi, Shojaei, Khodarahmi, and Mohammadi (2010) specified the best cultivars for each location by GGE biplot graphical method and identified three mega‐environments and concluded that Licord and SLM046 cultivars showed the highest and lowest genotyping reaction to the areas. In a study of four barley genotypes in eight regions in Turkey using the GGE biplot method, the study areas were divided into three mega‐environments (Kendal 2016) be using GGE biplot method determined that 8 locations can be divided into three mega‐environments. Two out of four genotypes had adequate general adaptability. Two other genotypes were specific adaptability in two of eight location. Sabaghnia, Dehghani, and Sabaghpour (2008) in a study for determination of mega‐environments in lentil regional choose three of seven environments.

The present study aimed to investigate the genotype–environment interaction and determine the most stable and most adaptable cultivars of sunflower in five different areas based on the GGE biplot method.

## 2 MATERIALS AND METHODS

Twelve sunflower hybrids were cultivated and evaluated within a randomized complete block design (RCBD) with three replications and five research stations including Karaj, Birjand, Firooz‐Abad, Kashmar, and Arak during two years (2016 and 2017). Tables 1 and 2 show the geographical specifications of research stations and names and origins of studied genotypes of sunflower, respectively.

**Table 1:** Geographical specifications of the experiment areas

Area	Longitude	Latitude	Elevation AMSL (m)	Average rainfall (mm)
Karaj	50°54'E	35°55'*N*	1,312	295
Birjand	59°12'E	32°52'*N*	1,491	171
Firooz‐Abad	52°36'E	29°32'*N*	1,484	337.8
Kashmar	58°48'E	35°53'*N*	1,109	178
Arak	49°46'E	34°06'*N*	1,708	341.7

**Table 2:** Names and code of sunflower varieties studied in the experiment

Genotype No.	Genotype	Origin	Genotype No.	Genotype	Origin
G1	Progress	Russia	G7	SHF81−90	Russia
G2	Gabur	Russia	G8	Zaria	Iran
G3	Zargol	Iran	G9	Favorite	Russia
G4	Armaverski	Russia	G10	Record	Romania
G5	Azargol	Iran	G11	Lakumka	Russia
G6	Master	Russia	G12	Bulg3	Bulgaria

Each experimental plot had 4 cultivation rows with a distance of 75 cm. Seeds were cultivated 10 cm away from each other, and all stages including irrigation and weeding were done manually and regularly. To delete the border effects, sampling was made on two middle rows. The amount of consuming nitrogen fertilizer was 100 kg/ha.

Before the combined analysis of variance, the variance homogeneity of experimental errors was examined by Bartlett's test. To determine the genotypes’ stability, the multivariate graphical technique of GGE biplot was used based on the decomposition to the following single values:

$$Y_{\mathrm{ij}}-\mu -\beta_{j}=\lambda_{1}\;\xi_{i1}\;\eta_{i1}+\lambda_{2}\;\xi_{i2}\;\eta_{i2}+\varepsilon_{\mathrm{ij}}$$

where *Y_ij_* is the average of *i*th genotype in *j*th environment; μ is the average of total genotypes; *β_j_* is the main effect of *j*th environment; *λ*_1_ and *λ*_2_ are the special values for first and second components; ξ_i1_ and ξ_i2_ are the special genotype vectors; η_j1_ and η_j2_ are the environmental vectors of first and second components; and *ε_ij_* is the remaining value for *i*th genotype in *j*th environment.

SAS v.9.1 software was used for Bartlett's test and combined variance analysis of data and GGE biplot based on five patterns: (a) determining the best genotype in each environment, (b) coordinates of average environment, (c) ranking the genotypes based on the ideal genotype, (d) ranking the environments based on the ideal environment, and (e) examining the relationship among the environments was used for graphical analysis.

## 3 RESULTS AND DISCUSSION

Variance homogeneity of experimental errors was examined using Bartlett's test of which the results confirmed the homogeneity of such errors. Hence, combined analysis of variance for grain yield was carried out as the results are shown in Table 3. Results indicated that the location, genotype, genotype × location effects are significant in 1% probability level. Significance of location effect revealed this fact that locations vary in terms of grain yield of genotypes and significance of genotype–location interaction shows that the genotypes’ yield varies from one location to another (Table 3). In this study genotype, location, year, year × location, genotype × location, genotype × year, and genotype × year×location proportion were 2.75%, 17.36%, 5.47%, 17%, 10.8%, 1.04%, and 7.48%, respectively. Grain yield of the cultivars ranged from 2.68 t/ha for *Zargol* to 3.34 t/ha for *Zaria*. In a study, Ullah et al. (2007) evaluated ten sunflower hybrids in seven environments in Pakistan for grain yield, and in this study, genotype, environment, and genotype × environment explain 2.59%, 81.89%, and 11.12% of variation, respectively. Haruna et al. (2017) in corn hybrids study for grain yield reported similar finding in multi‐environment trials where the largest proportion of total variation was attributed to environment and relatively smaller sources of variation to genotype. These researchers reported that genotype, genotype × environment, and environment proportion were 2.2%, 3.1%, and 94%, respectively. Jockovic *et al*. in the study of 24 sunflower hybrids in 12 environments reported that genotype, environment, and genotype–environment interactions proportion were 7.55%, 67.40%, and 25.05%, respectively (Jocković et al., 2019).

**Table 3:** Combined analysis of variance for grain yield of sunflower genotypes in 5 locations and 2 years

Source of variation	*df*	Sum of square	Mean square	% of total sums of squares
Location	4	88,809,433.47	22,202,358.37**	17.36
Year	1	28,011,177.12	28,011,177.12**	5.47
Location × Year	4	86,932,982.74	21,733,245.69**	17
Error	20	50,743,948.41	2,537,197.42	9.92
Genotype	11	14,074,860.90	1,279,532.81**	2.75
Location × Genotype	44	55,356,194.43	1,258,095.33**	10.8
Year × Genotype	11	5,350,765.33	486,433.21ns	1.04
Location × Year ×Genotype	44	38,295,766.78	870,358.34ns	7.48
Error	220	147,784,832.5	671,749.2	28.9
CV = 27.47%				

* ^, ^** and ns: Significant at 5%, 1% and not significant.

Results of GGE biplot method showed that the first and second main components are responsible for 50.6% and 22.8%, respectively, and in total, 73.4% of changes are explained by grain yield variance.

**Figure 1:**
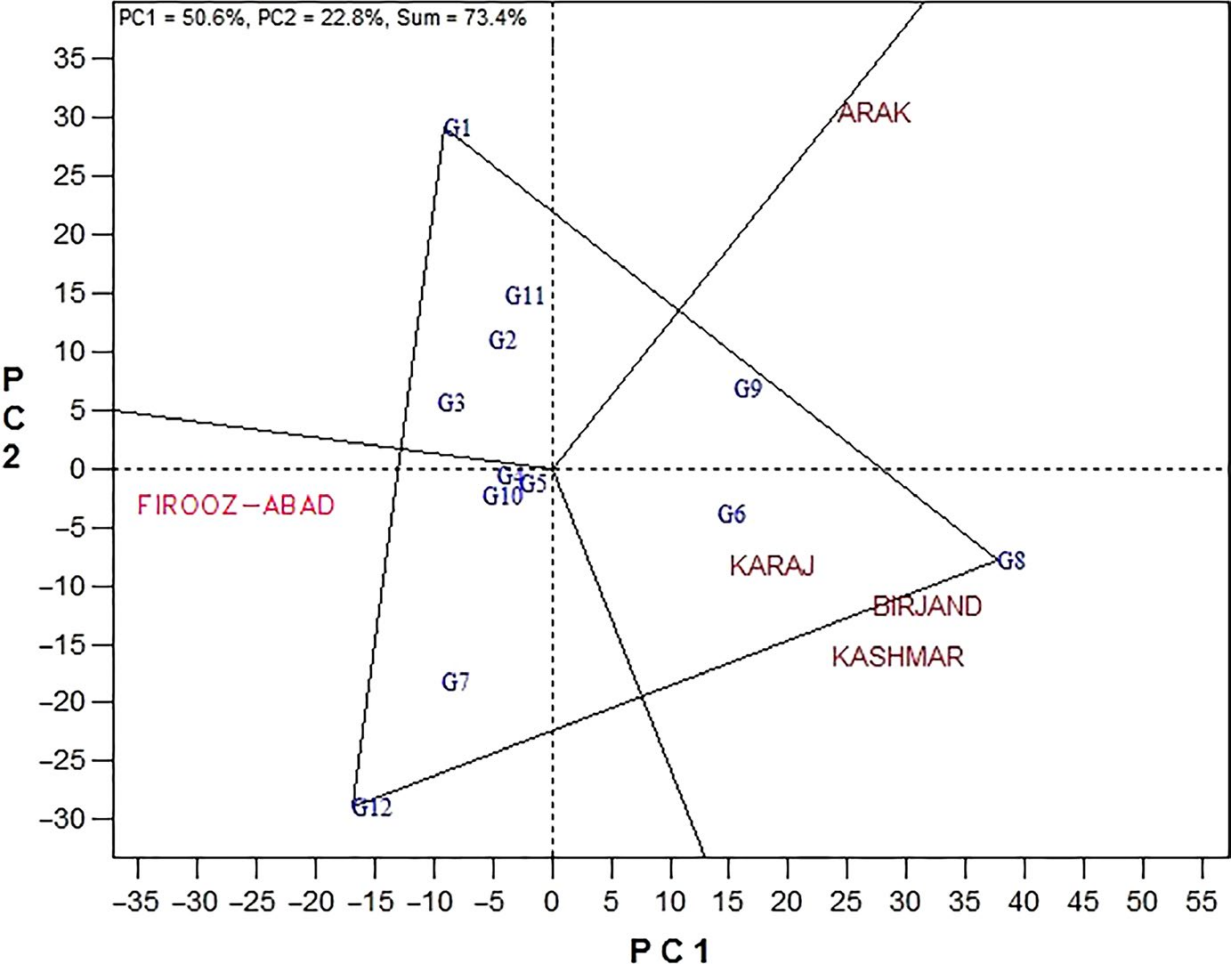
Polygon view of GGE biplot method for determining the appropriate cultivars in every environment. (G1: Progress, G2: Gabur, G3: Zargol, G4: Armaverski, G5: Azargol, G6: Master, G7: SHF81‐90, G8: Zaria, G9: Favorite, G10: Record, G11: Lakumka, G12: Bulg3)

To identify the best genotypes and mega‐environments, the GGE polygon view was plotted. Figure 1 shows a polygon scheme of 12 studied genotypes in 5 environments. As it is seen in this figure, genotypes with the most distance to the biplot center are linked together by lines and create a polygon; other genotypes are location inside the polygon. Then, lines are drawn perpendicularly on the sides of such polygon from the origin which makes distinct the mage‐environments (Yan et al., 2007). Based on this, genotypes Progress, Zaria, and Bulg3 are on the vertices of this polygon and called the best genotypes. Genotype Zaria showed the highest rate of yield in Birjand, Kashmar, and Karaj. In addition to this genotype, genotype Favorite and Master had high yield in order in these locations. In fact, these three genotypes yielded desirably over other genotypes in such locations. These three locations can be taken as a mega‐environment into account. Genotype Progress showed the highest rate of yield in Arak location, and as a result, this can be introduced as the best genotype in such environment. Genotypes Lakumka, Gabur, and Zargol yielded highly in this environment. Arak region was considered the second mega‐environment. Genotype Bulg3 showed highest yield in Firooz‐Abad followed by SHF81‐90. Since they are located on the center of biplot, genotypes Record, Azargol, and Armaverski were generally consistent with all environments. Firooz‐Abad environment was known as the third mega‐environment.

**Figure 2:**
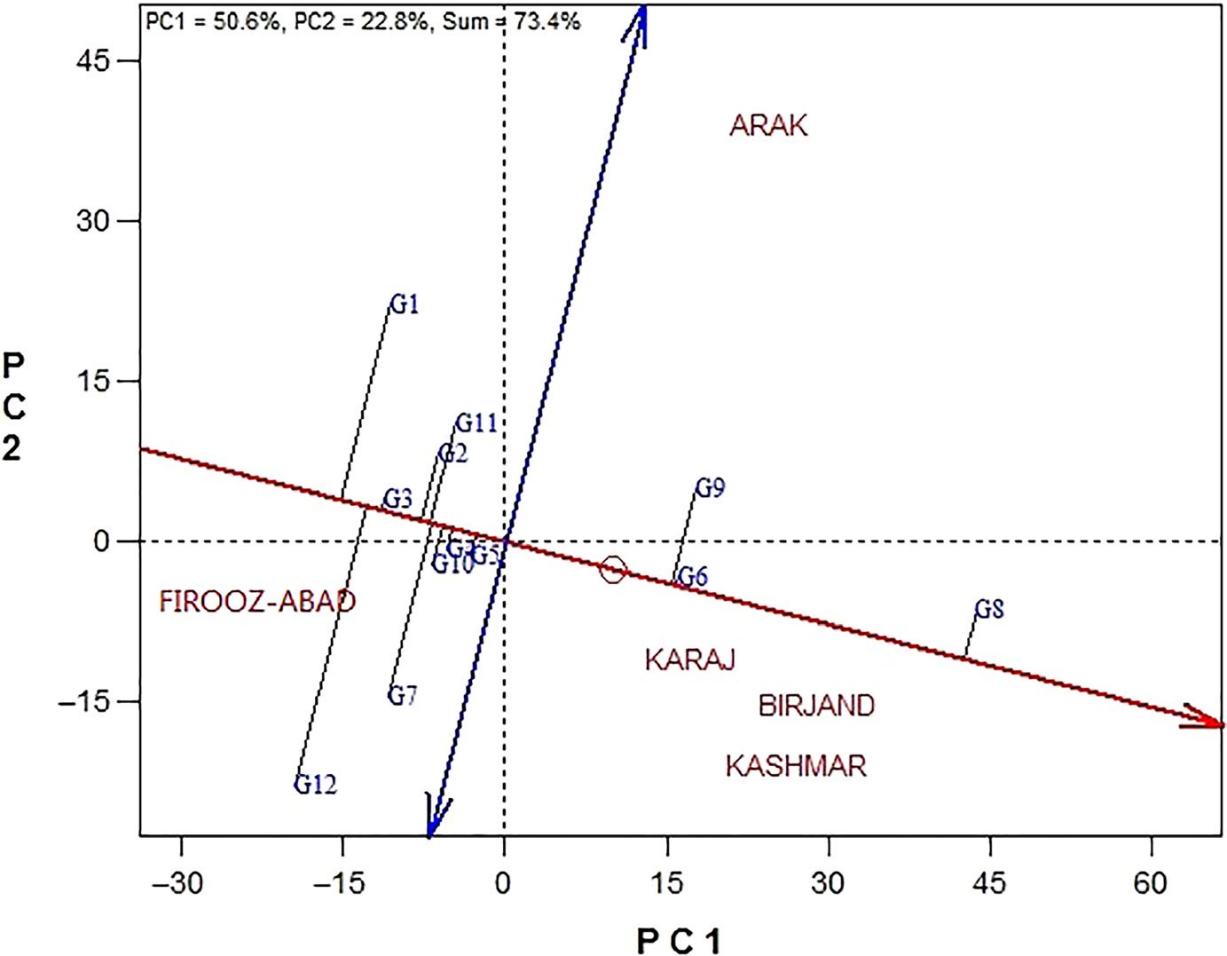
Simultaneous evaluation of grain yield and yield stability of the 12 sunflower genotypes in 5 environments by GGE biplot method (G1: Progress, G2: Gabur, G3: Zargol, G4: Armaverski, G5: Azargol, G6: Master, G7: SHF81‐90, G8: Zaria, G9: Favorite, G10: Record, G11: Lakumka, G12: Bulg3)

Figure 2 shows the genotypes’ ranking based on the grain yield and stability in 5 locations. Two‐dimensional graph of environments’ average coordinates is used to investigate the cultivars simultaneously on the basis of genotypes’ yield and stability. In this figure, a line that has an arrow and pass through a small circle (Mean of the environments) and also crosses the coordinate source is a criterion for evaluating the performance of the cultivars. The cultivar to the right of this axis has more yield. The line that is perpendicular to this line and has two arrows is a measure of the stability or instability of the cultivars. Any genotype closer to this line will be more stable (Yan, Hunt, Sheng, & Szlavnics, 2000). Therefore, genotype Master has the most stability, but it was weaker than the genotype Zaria in terms of grain yield. As a result, genotype Zaria was introduced as the best genotype. Genotype Progress with the most distance to the stability axis on the end left side of yield axis had the most instability and weakest yield of grain. Order of genotypes from the most desired to the most undesired in terms of grain yield is as follows: Zaria ˃ Master ˃ Favorite ˃ Azargol ˃ Armaverski ˃ Record ˃ SHF81‐90 ˃ Lakumka ˃ Gabur ˃ Zargol ˃ Bulg3 ˃ Progress.

**Figure 3:**
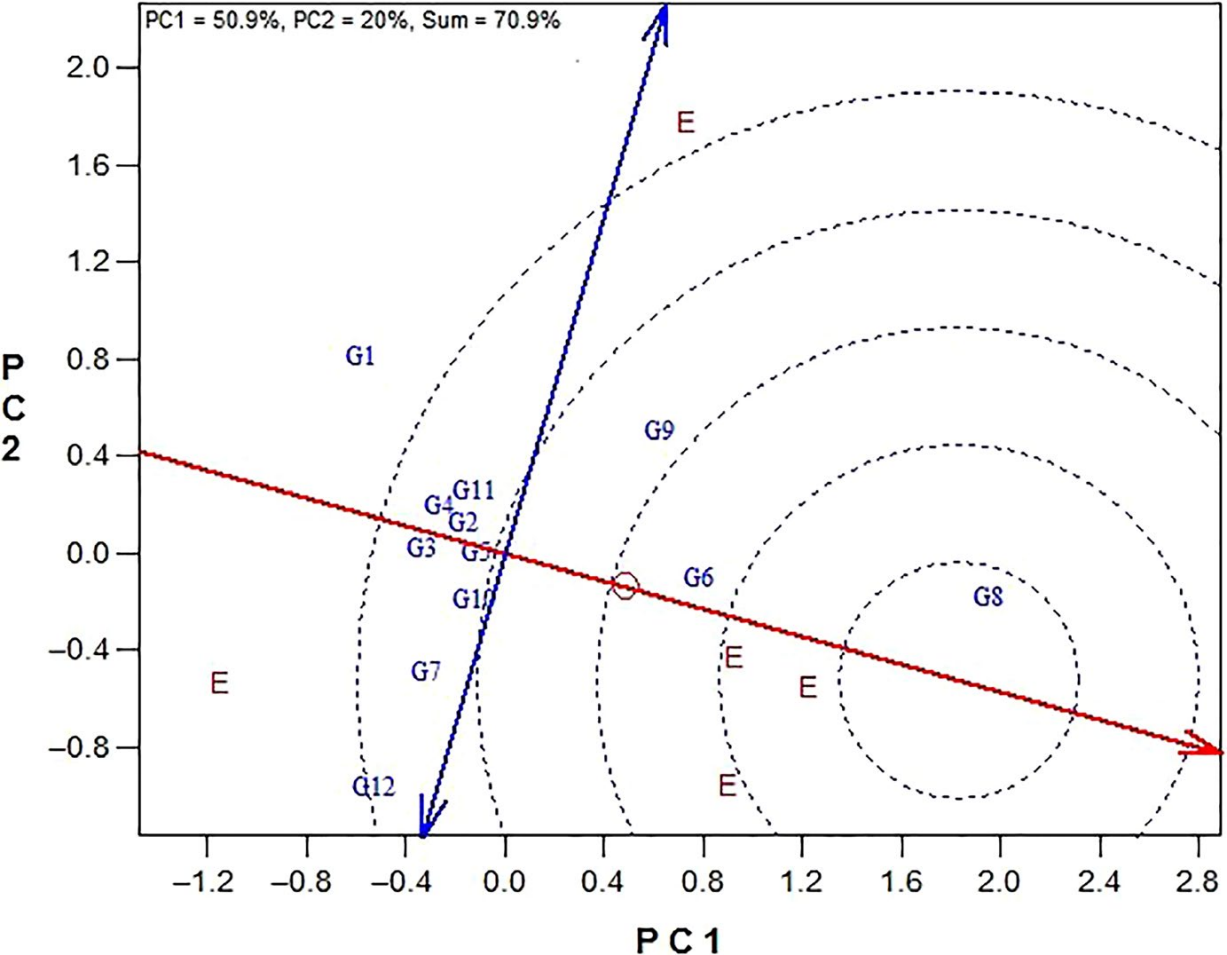
GGE biplot for ranking of the sunflower genotypes based on the ideal genotype. (G1: Progress, G2: Gabur, G3: Zargol, G4: Armaverski, G5: Azargol, G6: Master, G7: SHF81‐90, G8: Zaria, G9: Favorite, G10: Record, G11: Lakumka, G12: Bulg3)

Figure 3 is GGE biplot for ranking of the sunflower genotypes based on the ideal genotype. It is likely to identify the ideal genotype based on the concepts of high stability and yield. Based on this, that genotype is desired which has the most yield and maximum stability and any genotype with closest distance to this ideal genotype is known as the most desired one and the one with the most distance is introduced as most the undesired genotype (Yan & Kang, 2003). Based on this figure, Zaria genotype with minimum distance to the hypothetical ideal genotype was introduced as the best genotype and genotype Progress due to its furthest distance to the hypothetical ideal genotype is introduced as the most undesired genotype. Ranking of genotypes based on the ideal genotype from the most desired to most undesired genotypes is as follows: Zaria ˃ Master ˃ Favorite ˃ Record ˃ Azargol ˃ Gabur ˃ Lakumka ˃ Armaverski ˃ Zargol ˃ SHF81‐90 ˃ Bulg3 ˃ Progress (Figure 3).

**Figure 4:**
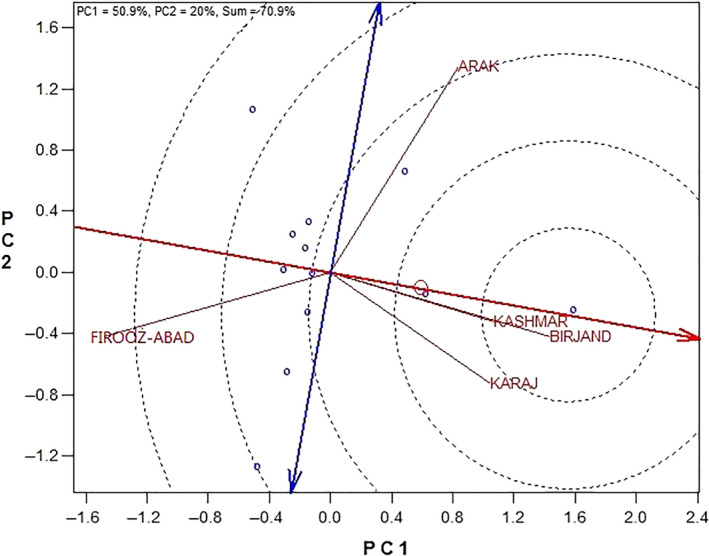
Ranking biplot for comparison of the environments with the ideal environment (G1: Progress, G2: Gabur, G3: Zargol, G4: Armaverski, G5: Azargol, G6: Master, G7: SHF81‐90, G8: Zaria, G9: Favorite, G10: Record, G11: Lakumka, G12: Bulg3)

Figure 4 is ranking biplot for comparison of the environments with the ideal environment. This figure identifies the most appropriate and most inappropriate environments. Based on this, the best environment is the one that has the closest distance from the ideal environment (concentric circles) and the most undesired one is the environment with the furthest distance to the ideal environment. Birjand and Kashmar were closest to the hypothetical ideal environment, as they were introduced as the best environments. Firooz‐Abad had the furthest distance to the hypothetical ideal environment which was introduced as the most undesired location. Order of location based on the hypothetical ideal environment from the most appropriate to most inappropriate locations is Birjand > Kashmar > Karaj > Arak > Firooz‐Abad (Figure 4).

**Figure 5:**
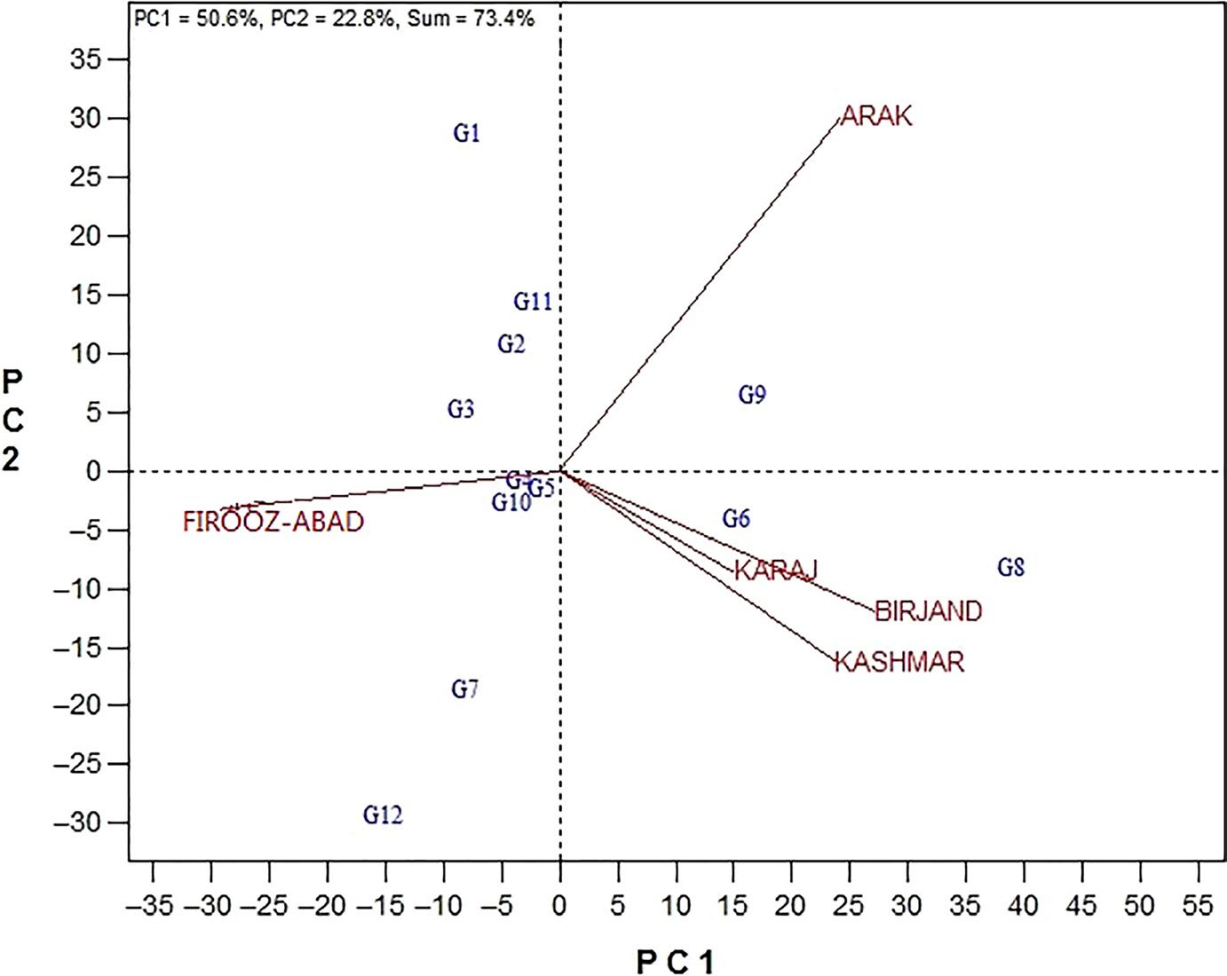
Biplot graph show relationship among locations in sunflower genotypes test. (G1: Progress, G2: Gabur, G3: Zargol, G4: Armaverski, G5: Azargol, G6: Master, G7: SHF81‐90, G8: Zaria, G9: Favorite, G10: Record, G11: Lakumka, G12: Bulg3)

The existing correlation among the environments can reveal the relationships among them and be used in future studies. Cosine of the angle between the environmental vectors indicates their correlation. If the angle between the two vectors is 180˚ the correlation is −1; the angle equal to 90° shows the correlation zero (lack of correlation) and when it is lower than 90°, there is positive correlation between two environments. By determining the correlation among the environments, similar environments can be identified and omitted in experiments on stability determination and cultivars stability for several years and several locations; this leads to the cost reduction (Yan & Kang, 2003;Yan & Rajcan, 2002). The angle of vectors for Karaj, Birjand, and Kashmar was <90° indicating positive correlations among them based on which they can be classified into one group. The angle of vectors for Karaj, Birjand, and Kashmar against the Arak was close to 90°. Therefore, they had a correlation of zero and close to zero. The angle between Arak and Firooz‐Abad vectors was higher than 90°; as a result, these locations are correlated negatively. This explains that two environments of Firooz‐Abad and Arak are separated from each other. Another important characteristic in relationship among the environments figure is the length of environment vector which is an estimation of standard deviation inside each environment and is also taken as an index for environments discrimination (Yan & Kang, 2003). Environment position showed that most of them have had long vectors indicating their high differentiability. Karaj environment has a vector shorter than other ones and hence is was less differentiated (Figure 5).

## 4 CONCLUSION

In this research, a significant variation was observed between the cultivars for grain yield and it was determined that the biplot can be a useful method for the discrimination of genotypes and different environments. Results revealed that the Zaria genotype had the highest grain yield and the highest yield stability. This method makes it possible to differentiate factors that affect different grain environments. These results determined which environment is more capable for detecting the best genotype. This method can help the breeders to make a more accurate decision about the release of a suitable cultivar; in this technique, visual and quantitative evaluations can be very useful for detection of performance and stability of the cultivars. Analysis of sunflower cultivars based on grain yield in different environments showed that it is possible to identify the cultivars with both high grain yield and stability for each location. The results showed that evaluation of different genotypes in different locations is not only useful in selecting high‐yielding and stable cultivars but also it can be useful for determining the appropriate cultivars for low‐yielding environments (Jocković et al., 2019). These results can be very useful for selecting parents for future breeding programs. The location of Birjand, Kashmar, and Karaj in one mega‐environment indicates that one of these environments can be used for future evaluations, and climatic conditions also confirm this.

## CONFLICT OF INTEREST

The authors have declared no conflict of interest.

## ETHICAL APPROVAL

This study does not involve any human or animal testing.

## INFORMED CONSENT

Written informed consent was obtained from all study participants.
